# Retraction: Epstein-Barr Virus Nuclear Antigen 3C Stabilizes Gemin3 to Block p53-mediated Apoptosis

**DOI:** 10.1371/journal.ppat.1009556

**Published:** 2021-04-30

**Authors:** The PLOS Pathogens Editors

Following the publication of this article [1], concerns were raised regarding the results presented in Figures 1, 3, 4, 5, and 6. Specifically:

- Vertical discontinuities have been observed in the following panels:
  - Figure 1B, WB:E3C panel: between lanes 3–4, 5–6, and 8–9.
  - Figure 1B, WB:Germin3 panel: between lanes 2–3, 3–4, 4–5, and 8–9.
  - Figure 3D, S^35^-Germin3 panel: between lanes 3–4 and 5–6.
  - Figure 4B, WB:Germin3 panel: between lanes 1–2.
  - Figure 5B, S^35^-p53 panel: between lanes 3–4 and 5–6.
  - Figure 5C, WB:Germin3 panel: between lanes 5–6.
- In Figure 3C the 621–992 panel appears similar to the 1–992 panel when flipped. The authors agreed that the panels are identical and indicated that the wrong panel was inadvertently used for the 621–992 results during figure preparation.
- In the Comassie Staining panel of Figure 3C, irregularities have been detected in the signal displayed between the 95 and 72 markers of the GST-Gemin3 lane. The authors indicate that the wrong image was uploaded for this panel and the original uncropped blot underlying this panel confirms that signal has been obscured in the published panel.
- Repetitive bands have been observed within lane 2 and between lane 2 and lane 4 of the Figure 3D Comassie Staining panel. The authors confirm that this image was compiled incorrectly, and the original uncropped blot underlying this panel does not match the published figure.
- In Figure 6B, there appears to be horizontal and vertical discontinuities surrounding the band presented in lane 4 of the WB:Germin3 panel. The authors were unable to provide the original uncropped blot underlying this panel.

The authors have provided individual level data underlying the graphs presented in Figures 3, 4, 5, and 6. Due to the time elapsed since the experiments were conducted the authors were unable to provide the original uncropped blots underlying the Figure 1B WB:E3C panel, the Figure 3D S^35^-Germin3 panel, the Figure 5B S^35^-p53 panel, the Figure 5C WB:Germin3 panel, and the Figure 6B WB:Germin3 panel. Underlying blots were provided for the Figure 1B WB:Germin3 panel and the Figure 4B WB:Germin3 panel. The blot provided for the Figure 1B WB:Germin3 panel did not support all the results presented in the published figure, in particular those presented in lanes 2, 5, and 8. The blot underlying the Figure 4B WB:Germin3 panel, although of low resolution, appears to support the published results.

In light of the concerns affecting multiple figure panels that question the integrity of these data, the *PLOS Pathogens* Editors retract this article.

ESR and QC agreed with the retraction, but stand by the article's findings. YG, BX, SB, AS, JL, and TG either did not respond directly or could not be reached.
